# Complete genome sequence of *Thermus scotoductus* AK-1 isolated from an Arctic hydrothermal site in Alaska

**DOI:** 10.1128/mra.00419-26

**Published:** 2026-06-04

**Authors:** Seung Chul Shin, Seon-hui Yu, Sung-Jong Jeon, Han-Woo Kim

**Affiliations:** 1 Division of Life Sciences, Korea Polar Research Institute (KOPRI) 123591, Incheon, Republic of Korea; 2 Department of Polar Sciences, University of Science and Technology, Incheon, Korea; 3 Department of Biomedical Engineering, Dong-Eui University 34938, Busan, Republic of Korea; 4 Department of Smart-Biohealth, Dong-Eui University 34938, Busan, Republic of Korea; 5 Smart Bio-Health Institute, Dong-Eui University, Busan, Republic of Korea; Nanchang University, Nanchang, Jiangxi, China

**Keywords:** Thermus scotoductus, Arctic hydrothermal site, Alaska

## Abstract

We report the complete genome sequence of *Thermus scotoductus* AK-1 strain isolated from an Arctic hydrothermal site in Alaska. The chromosome was sequenced using the PacBio Revio HiFi platform. The complete circular genome sequence is 2.21 Mb in length.

## ANNOUNCEMENT

*Thermus scotoductus* AK-1 was isolated from an Arctic hydrothermal site in Alaska (65°05′24.8″N 164°55′22.6″W), where the *in situ* temperature ranged from 74.8°C to 75.8°C. *Thermus scotoductus* is a thermophilic, aerobic heterotroph belonging to the phylum Deinococcota, one of the most ancient bacterial lineages. Strains of this species are generally Gram-negative and have been isolated from hydrothermal environments at 55°C to 70°C (1, 2). *T. scotoductus* SA-01 is notable for its ability to grow under microwave radiation (3). To provide a genomic resource for comparative studies of thermophilic bacteria inhabiting polar geothermal environments, we report the complete genome sequence of *T. scotoductus* AK-1.

To isolate the microorganism, a 10 mL water sample was supplemented with 0.1% yeast extract and incubated at 70°C for 2 days for enrichment. The turbid culture was streaked onto TYE medium (0.5% tryptone, 0.1% yeast extract, 0.1% NaCl, 0.02% MgSO_4_·7H_2_O, 0.005% CaCl_2_·2H_2_O, and 0.05% KCl), solidified with 0.7% Gelrite, and incubated at 70°C for 2 days. A single colony that appeared on the plate was re-streaked onto a TYE plate and incubated at 70°C for 2 days to obtain a pure isolate.

Genomic DNA was extracted from 3-day TYE broth cultures using the Wizard HMW DNA Extraction Kit (Promega, Madison, WI, USA). The isolate was identified by 16S ribosomal RNA (rRNA) gene sequencing using primers 27F and 1492R (4), followed by sequencing on an ABI 3730 Genetic Analyzer (Applied Biosystems, USA) at MACROGEN (Seoul, Republic of Korea). The 16S rRNA gene sequence showed 99.41% identity to *T. scotoductus* SA-01 (GCF_000187005.1) based on the National Center for Biotechnology Information Basic Local Alignment Search Tool analysis (1).

Genomic DNA was quantified and sized using a Qubit fluorometer (Thermo Fisher Scientific, DE, USA) and a Femto Pulse system (Agilent, CA, USA). Without prior shearing, 10 μg of DNA was used to construct a high-fidelity (HiFi) library using the SMRTbell Prep Kit 3.0 (PacBio, CA, USA). After library quality control using the same instruments, fragments > 10 kb were size-selected using the BluePippin system (Sage Science, USA). SMRT sequencing was performed on a PacBio Revio system at PHYZEN Co. (Seongnam, Republic of Korea). Base calling, adapter removal, and HiFi read filtering were conducted using SMRT Link (v25.3) with default parameters. A total of 167,445 HiFi reads, comprising approximately 1.1 Gb with an average read length of 6,386 bp and an N50 of 7,096 bp, were obtained.

Genome assembly was performed using Flye (v2.9.6), resulting in a single circular genome of 2,212,688 bp with a GC content of 65.2%. Circularity was validated by inspecting soft-clipped reads at the contig ends using Minimap2 (v2.30) (5), SAMtools (v1.23) (6), and Integrative Genomics Viewer (v2.16) (7). Genome annotation was carried out using the Prokka (v1.2.0) pipeline (8), which incorporates Prodigal (v2.60) (9), Aragorn (v1.2.41) (10), and RNAmmer (v1.2) (11). A total of 2,274 coding DNA sequences, 50 transfer RNA genes, and two rRNA operons were identified in *T. scotoductus* AK-1. The completeness of the annotated genome was assessed using BUSCO (v6.0.0) (12), and *T. scotoductus* AK-1 showed 97.3% completeness against the thermus_odb12 data set.

## Data Availability

The genome sequence has been deposited in GenBank under accession number JBWUIR000000000 and BioProject accession number PRJNA1446209. The raw sequencing reads from PacBio HiFi system have been deposited under accession number SRR37901017.

## References

[1] Gounder K, Brzuszkiewicz E, Liesegang H, Wollherr A, Daniel R, Gottschalk G, Reva O, Kumwenda B, Srivastava M, Bricio C, Berenguer J, van Heerden E, Litthauer D. 2011. Sequence of the hyperplastic genome of the naturally competent Thermus scotoductus SA-01. BMC Genomics 12:577. doi:10.1186/1471-2164-12-577 22115438 PMC3235269

[2] Woese CR. 1987. Bacterial evolution. Microbiol Rev 51:221–271. doi:10.1128/mr.51.2.221-271.1987 2439888 PMC373105

[3] Cockrell AL, Fitzgerald LA, Cusick KD, Barlow DE, Tsoi SD, Soto CM, Baldwin JW, Dale JR, Morris RE, Little BJ, Biffinger JC. 2015. Differences in physical and biochemical properties of Thermus scotoductus SA-01 cultured with dielectric or convection heating. Appl Environ Microbiol 81:6285–6293. doi:10.1128/AEM.01618-15 26150459 PMC4542235

[4] Frank JA, Reich CI, Sharma S, Weisbaum JS, Wilson BA, Olsen GJ. 2008. Critical evaluation of two primers commonly used for amplification of bacterial 16S rRNA genes. Appl Environ Microbiol 74:2461–2470. doi:10.1128/AEM.02272-07 18296538 PMC2293150

[5] Li H. 2018. Minimap2: pairwise alignment for nucleotide sequences. Bioinformatics 34:3094–3100. doi:10.1093/bioinformatics/bty191 29750242 PMC6137996

[6] Danecek P, Bonfield JK, Liddle J, Marshall J, Ohan V, Pollard MO, Whitwham A, Keane T, McCarthy SA, Davies RM, Li H. 2021. Twelve years of SAMtools and BCFtools. Gigascience 10:giab008. doi:10.1093/gigascience/giab008 33590861 PMC7931819

[7] Robinson JT, Thorvaldsdóttir H, Winckler W, Guttman M, Lander ES, Getz G, Mesirov JP. 2011. Integrative genomics viewer. Nat Biotechnol 29:24–26. doi:10.1038/nbt.1754 PMC3346182 21221095

[8] Seemann T. 2014. Prokka: rapid prokaryotic genome annotation. Bioinformatics 30:2068–2069. doi:10.1093/bioinformatics/btu153 24642063

[9] Hyatt D, Chen GL, LoCascio PF, Land ML, Larimer FW, Hauser LJ. 2010. Prodigal: prokaryotic gene recognition and translation initiation site identification. BMC Bioinformatics 11:111 doi:10.1186/1471-2105-11-119 20211023 PMC2848648

[10] Laslett D, Canback B. 2004. ARAGORN, a program to detect tRNA genes and tmRNA genes in nucleotide sequences. Nucleic Acids Res 32:11–16. doi:10.1093/nar/gkh152 14704338 PMC373265

[11] Lagesen K, Hallin P, Rødland EA, Staerfeldt H-H, Rognes T, Ussery DW. 2007. RNAmmer: consistent and rapid annotation of ribosomal RNA genes. Nucleic Acids Res 35:3100–3108. doi:10.1093/nar/gkm160 17452365 PMC1888812

[12] Simão FA, Waterhouse RM, Ioannidis P, Kriventseva EV, Zdobnov EM. 2015. BUSCO: assessing genome assembly and annotation completeness with single-copy orthologs. Bioinformatics 31:3210–3212. doi:10.1093/bioinformatics/btv351 26059717

